# Pericytes as mediators of infiltration of macrophages in multiple sclerosis

**DOI:** 10.1186/s12974-021-02358-x

**Published:** 2021-12-24

**Authors:** Deepak Kumar Kaushik, Anindita Bhattacharya, Brian Mark Lozinski, V. Wee Yong

**Affiliations:** 1 Hotchkiss Brain Institute and Department of Clinical Neurosciences, University of Calgary, 3330 Hospital Drive, Calgary, AB T2N 4N1 Canada; 2 Division of Biomedical Sciences, Faculty of Medicine, Memorial University of Newfoundland, 300 Prince Philip Dr, St. John’s, NL A1B3V6 Canada

**Keywords:** Pericyte, Multiple sclerosis, CSPGs, Inflammation, Brain, Macrophage

## Abstract

**Background:**

Multiple sclerosis (MS) is a neurodegenerative condition of the central nervous system (CNS). It is associated with blood–brain barrier (BBB) breakdown and intravasation of leukocytes, particularly monocyte-derived macrophages, into the CNS. Pericytes are mural cells that are encased within the basement membrane of vasculature, and they contribute functionally to the neurovascular unit. These cells play an important role in maintaining BBB integrity and CNS homeostasis. However, the critical role of pericytes in mediating inflammation in MS or its models is unclear. Whether pericytes infiltrate into the CNS parenchyma in MS also needs clarification.

**Methods:**

CNS samples from the experimental autoimmune encephalomyelitis (EAE) mouse model of MS were collected at different time points for immunohistochemical analysis of pericytes along the inflamed vasculature. These findings were validated using MS brain specimens, and further analysis of pericyte involvement in inflammation was carried out by culturing primary pericytes and macrophages. Multiplex ELISA, transmigration assay and real-time PCR were used to study the inflammatory potential of pericytes in cultures.

**Results:**

We found that pericytes exhibit a heterogenous morphology, with notable elongation in the inflamed perivascular cuffs of EAE. This was manifested by a decrease in pericyte density but an increase in the coverage by pericytes along the vasculature. Chondroitin sulfate proteoglycans (CSPGs), a family of extracellular matrix proteins enriched within inflamed perivascular cuffs, elevated levels of pro-inflammatory chemokines/cytokines in pericytes in culture. Importantly, pericytes stimulated with CSPGs enhanced macrophage migration. We did not detect pericytes in the CNS parenchyma during EAE, and this was corroborated in MS brain samples.

**Conclusions:**

Our data suggest that pericytes seek to restore the BBB through increased coverage, but that their exposure to CSPGs prompt their facilitation of macrophages to enter the CNS to elevate neuroinflammation in EAE and MS.

**Supplementary Information:**

The online version contains supplementary material available at 10.1186/s12974-021-02358-x.

## Introduction

MS is a chronic inflammatory-neurodegenerative condition that affects the central nervous system (CNS). Blood–brain barrier (BBB) dysregulation and leukocyte migration into the CNS, followed by demyelination and neuroaxonal loss, are recognized as some of the hallmarks of MS pathology [1–3]. However, the regulation of leukocyte transmigration into the CNS, the majority of which are monocyte-derived macrophages, and the specific mechanisms by which they traverse the BBB remain to be fully defined. Pericytes are contractile cells that contact the endothelial layer of capillaries and post-capillary venules throughout the body and they are encased within the basement membranes that line vessels [4, 5]. Pericytes, along with the endothelium, astrocytes and neurons, constitute the neurovascular unit (NVU) and help maintain BBB integrity [6]. In this regard, it is proposed that pericytes’ dysfunction along the vasculature in the CNS is correlated to BBB permeability [7]. Notably, the brain has the highest ratio of pericytes to endothelial cells, which highlights the significance of pericytes in contributing to CNS homeostasis [5]. Indeed, in disease states including Alzheimer’s disease, stroke, spinal cord injury, and diabetic retinopathy, pericyte dysfunction is associated with an increase in vascular permeability, scar formation after acute injury, tight junction degradation, and BBB disruption [8–10].

A recent study shows that pericyte-deficient (*Pdgfb*^*ret/ret*^) adult mice have increased transmigration of leukocytes into the brain resulting in enhanced disease severity of experimental autoimmune encephalomyelitis (EAE), an inflammatory model of MS [11]. While the study infers the functions of pericytes in a genetically deficient model, it does not address the role of intact pericytes in the event of EAE/MS pathology, and how wild-type pericytes in EAE may mediate neuroinflammation. Another study reports an increase in the number of pericytes in active MS lesions over chronic lesions [12], but the clinical relevance of this is not understood. Other studies suggest that extracellular matrix (ECM) components including fibronectin and collagen-I may influence pericyte morphology, migration, and proliferation, while heparan sulfate proteoglycans have inhibitory effects [13]. In this regard, the influence of another major ECM component, chondroitin sulphate proteoglycans (CSPGs), and their dynamic interactions with pericytes with regard to their influence on BBB integrity, inflammatory responses, and facilitating leukocyte migration are not explored. This is an important question since CSPGs are highly expressed in MS lesions and they inhibit oligodendrocyte precursor cell (OPC) differentiation [14]. CSPGs also stimulate the production of pro-inflammatory chemokines/cytokines in macrophages, thereby facilitating their migration [15, 16]. Thus, in this study, we have sought to study the activity of pericytes in EAE and their response to CSPGs. We have focused on inflammatory perivascular cuffs of post-capillary venules [17], a CSPG-enriched space where leukocytes particularly monocytes gather prior to entering the CNS parenchyma. Our results highlight the contribution of CSPG–pericyte interactions in facilitating macrophage infiltration into the parenchyma in EAE/MS.

## Materials and methods

### EAE induction

Animal experiments were conducted in accordance with Canadian Council on Animal Care guidelines and with ethics approval from the University Animal Care Committee. Briefly, 8- to 10-week-old female C57BL/6 mice (Charles River) were immunized with 50 μg/100 μL of myelin oligodendrocyte glycoprotein (MOG) 35–55 peptide (Protein and nucleic acid facility, Stanford, CA) in complete Freund’s adjuvant supplemented with 4 mg/mL heat-inactivated *Mycobacterium tuberculosis* H37Ra (Fisher scientific, Toronto, Canada) subcutaneously. On days 0 and 2 post-MOG immunization, pertussis toxin (300 ng) was injected intraperitoneally. Animals were monitored daily for clinical signs of EAE on the 15-point scale, as described by Weaver et al.[18]. Cerebellar and spinal cord tissues were collected on day 10–13 (pre-peak of EAE), day 16 (peak clinical severity), and day 21 and day 35 (post-peak EAE) of EAE time course for immunohistochemistry. Cerebellum from naïve animals served as experimental controls.

### MS brain tissues

Frozen brain tissues from chronic cases of MS were obtained from the UK MS Tissue Bank at Imperial College, London (www.ukmstissuebank.imperial.ac.uk; provided by Richard Reynolds and Djordje Gveric) and Dr. Alex Prat (University of Montreal). Two of the samples were diagnosed as having secondary progressive MS: a 60-year-old female (Fig. 6A) and a 61-year-old male (Fig. 6B), and one was 26-year-old male diagnosed as relapsing–remitting MS (Fig. 6C). Brain tissue sections from cortical areas were analyzed for this study.

### Tissue processing, immunohistochemistry and confocal microscopy

Mice were perfused with phosphate-buffered saline (PBS) and tissues were harvested and frozen in optimal control temperature medium (VWR, 95057-838) and stored at − 80 °C until cryosectioning. 20-μm-thick sections were cut using a cryostat followed by immunohistochemistry. For this, EAE cerebellum tissues and MS brain sections were fixed with ice-cold methanol, or with 4% PFA followed by 0.2% Triton X-100, and blocked with 3% BSA before staining with the following primary antibodies: neural/glial antigen 2 (NG2) (Millipore, AB5320, 1:200) and platelet derived growth factor receptor beta (PDGFRβ) (Invitrogen, 16-1420-82, 1:100; R&D Systems, AF385, 1:100), which were used as the primary markers of pericytes. Anti-pan laminin (a kind gift from Dr. L. Sorokin, Westfälische Wilhelms-Universität, Münster, Germany; 1:1000) that stains the basement membranes of post-capillary venules, and anti-CD31, an endothelial marker (Abcam, 28364, 1:50) were used to characterize post-capillary venules and perivascular cuffs. Anti-CD45 (BD Pharmingen, 550539, 1:75) and anti-F4/80 (Biorad, MCA497RT, 1:100) were used for pan-leukocytes and myeloid cells, respectively. Nuclei were visualized with nuclear yellow (Hoechst). Confocal images were acquired in Z-stacks with ‘confocal-in-a-box’ (Olympus Fluoview FV10i confocal microscope) using a 60× oil-immersion objective.

### Quantification: pericyte coverage ratio and density

The pericyte coverage ratio along the blood vasculature in cerebellar sections was quantified at three time periods in the EAE model (day 10–13, day 16, and day 21) and in naïve animals. Confocal images from cerebellar tissues stained to visualize pericytes, using the markers NG2 and PDGFRβ, and blood vessels through CD31, were assessed. Three naïve and three EAE animals were examined at each time point and three fields of view were quantified per animal. The pericyte coverage ratio was quantified by measuring the total length of the blood vessels in each field of view and the total length of the blood vessel covered by pericytes in each field of view. The pericyte coverage ratio in 60× confocal images having an area of 215 μm × 215 μm was calculated as follows:

$$\frac{{{\text{Length}}\;{\text{of}}\;{\text{blood}}\;{\text{vessels}}\;{\text{covered}}\;{\text{by}}\;{\text{pericytes}} \; \left( {\upmu {\text{m}}} \right)}}{{{\text{Total}}\;{\text{length}}\;{\text{of}}\;{\text{blood}}\;{\text{vessels}} \;\left( {\upmu {\text{m}}} \right)}}.$$

To quantify pericyte density, the number of pericytes along the blood vasculature was quantified in each 60× confocal image, having an area of 215 μm × 215 μm.

### Primary pericyte cultures in vitro

Mouse brain vascular pericytes (iXCells Biotechnologies, 10MU-014) were grown in cell culture flasks coated in 0.01% poly-l-lysine (PLL, Trevigen, 3438-100-01). Cells were grown in Supplemented Mouse Pericyte Growth Media (SMPGM), i.e., 2% fetal bovine serum (FBS), 1% penicillin/streptomycin, and 1% Pericyte Growth Supplement (iXCells, Biotechnologies, MD-0092) in an incubator at 37 °C and 5% CO_2_. Pericytes were plated in PLL-coated black 96-well plates (BD Falcon, 353219) at a density of 7500 cells per well in 200 μL of SMPGM. 48 h later, the medium was replaced with SMPGM containing 0.2% FBS. For stimulation, pericytes were either treated with an inflammatory cytokine cocktail of recombinant mouse interferon gamma (IFN-γ) (PeproTech, 345-05, 10 ng/mL) and recombinant mouse IL-1β (R&D, 401-ML/CF, 10 ng/mL), or with CSPGs (Millipore, CC117, 10 μg/mL). After 48 h of treatment, the medium was discarded, and the cells were then overlaid with fresh serum-free DMEM for another 24 h to eliminate the presence of inflammatory stimuli from the culture medium. Each condition was carried out with four technical replicates. Post-24 h, the conditioned media were collected and centrifuged at 2000 rpm for 3 min to pellet any floating cells. The conditioned media were then stored at − 80 °C for analysis.

### Bone marrow-derived macrophage isolation

Bone marrows from euthanized C57BL/6 mice were flushed out using Dulbecco’s modified Eagle medium (DMEM) (Sigma, D5671) and centrifuged at 1200 rpm for 10 min. The pellet was resuspended in complete high glucose bone marrow growth medium (DMEM, 10% FBS, 2% penicillin/streptomycin and 10% supernatant from the L929 cell-line) and seeded at a density of 10^7^ on 10 cm petri dishes as previously described [16]. Cells were grown at 37 °C in 8.5% CO_2_ for 5 days, after which half the media was replaced with fresh media. At day 7, the full media was changed. Cells were used after 7 days of culture.

### TNFα and MMP9 enzyme-linked immunosorbent assay (ELISA) and Luminex assay

Conditioned media collected from control and treated pericytes were assessed for TNFα concentrations using an ELISA as per manufacturer’s instructions (Invitrogen, BMS607-3). The remaining conditioned media from the control and treated pericytes were assessed using a mouse 31-plex chemokine/cytokine Luminex assay (Eve Technologies, MD31). MMP9 ELISA was carried out using the pro-MMP9 ELISA kit on supernatants from untreated and CSPG-treated bone marrow-derived macrophages (BMDMs) (Invitrogen).

### Boyden chamber transmigration assay

Pericytes were plated in 6-well culture plates at a density of 5 × 10^5^. Twenty-four hours later, the medium was replaced with SMPGM containing 0.2% FBS. Pericytes were either treated with an inflammatory cytokine cocktail of recombinant mouse IFN-γ and recombinant mouse IL-1β, mixture of CSPGs or lipopolysaccharide (LPS) (*E. coli*, 055:B5, Sigma, L5418, 10 ng/mL) as a positive control. Three days later, the treated medium was replaced with SMPGM containing 0.2% FBS. Forty-eight hours later, BMDMs were seeded at a density of 2 × 10^5^ cells per transwell filter insert with 5.0 μm pores (Corning Costar, 3421) in serum-free DMEM. The conditioned medium from the control or treated pericytes was placed in the bottom compartment of the Boyden chamber, to potentially serve as a chemotactic stimulus for the BMDMs. Twenty-two hours later, the filters were washed with PBS to remove any remaining cells. The filters were then fixed and stained with hematoxylin, Gill #2 (Sigma, GHS216). The number of BMDMs that migrated was assessed using a 20× brightfield microscope (Olympus BX51). The number of BMDMs that migrated were averaged for each filter by assessing 4 fields of view around the center of the filter, while the edges of the filter were excluded. Images were blinded before quantification.

### Real-time PCR

Quantitative PCR was performed on RNA isolated from treated pericytes to study the changes in pro-MMP9 transcripts. For this, the cells were treated with an inflammatory cocktail or CSPGs for 6 h and then lysed for RNA isolation by Trizol method. Pro-MMP9 primers were purchased from Qiagen.

### Statistics

Datasets were tested for normal distribution using the Shapiro–Wilk normality test (*P* > 0.05). Multiple groups were compared using a one-way ANOVA with Tukey’s multiple comparison post hoc test, where *P* < 0.05 was considered significant. All quantified results are stated in the form of mean ± SD. All statistical analyses were performed with Prism 8.0 software (GraphPad).

## Results

### Pericyte dynamics during EAE pathology

**Fig. 1:**
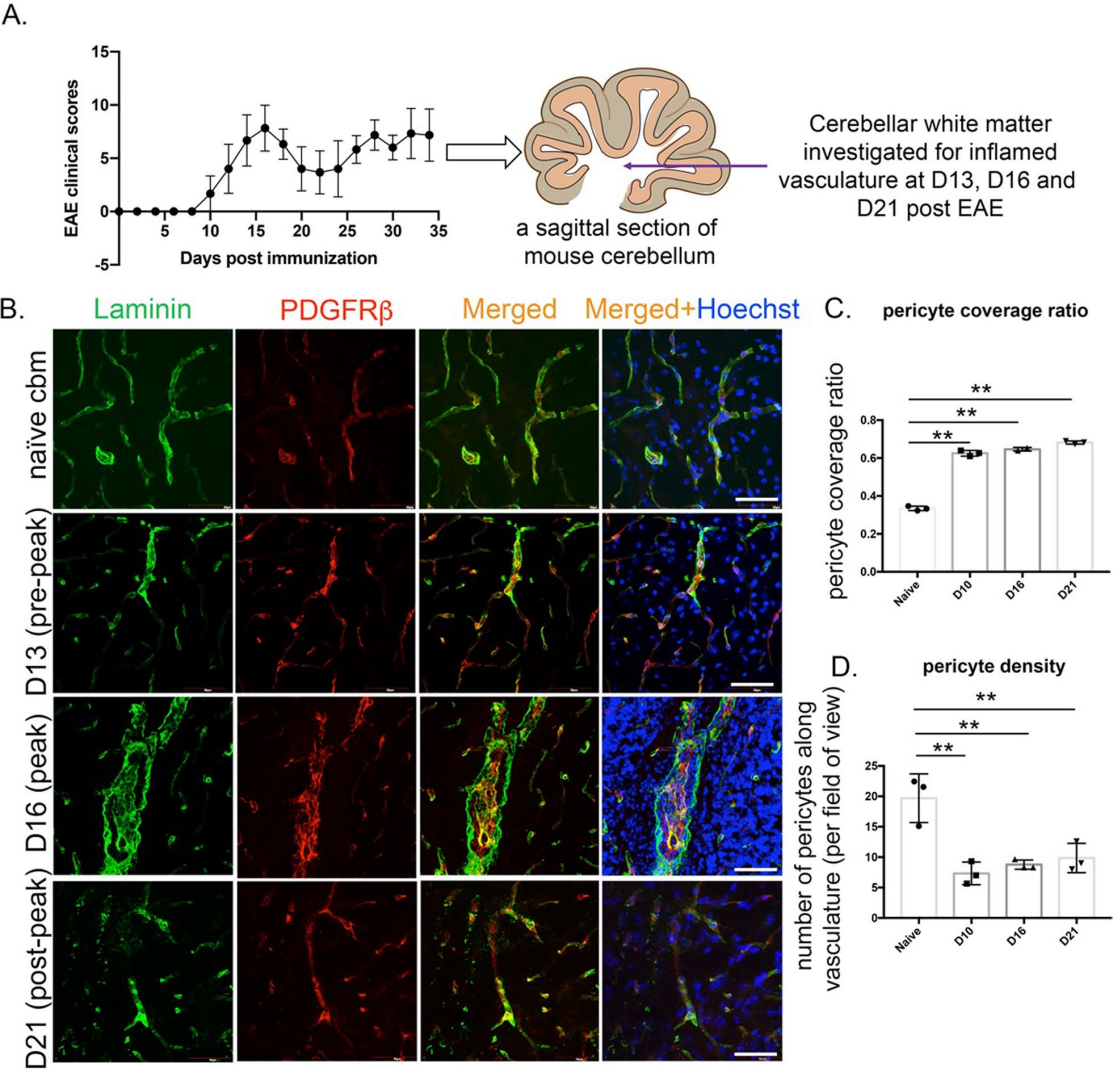
Pericyte heterogeneity in EAE pathology. **A** The graph represents clinical scores of the three EAE mice that were investigated for this study. The cartoon shows white matter within the cerebellum sagittal sections studied for pericytes. **B** Confocal images of capillary basement membranes delineated with anti-laminin antibodies exhibit PDGFRβ+ pericytes encased within laminin+ basement membranes in pre-peak (D13), peak (D16) and post-peak (D21) EAE cerebellum. Scale 50 µm. **C** Pericyte coverage ratio as described by the total length of pericytes along the vasculature shows increase in each of the EAE time points observed as compared to the naïve cerebellar pericytes. **D** Pericyte density as studied by the nucleated pericytes abutting the length of the vasculature in the given field and represented as number of cells per field of view. Scale 100 µm. Datasets are expressed as mean ± SD. **p < 0.01

**Fig. 2:**
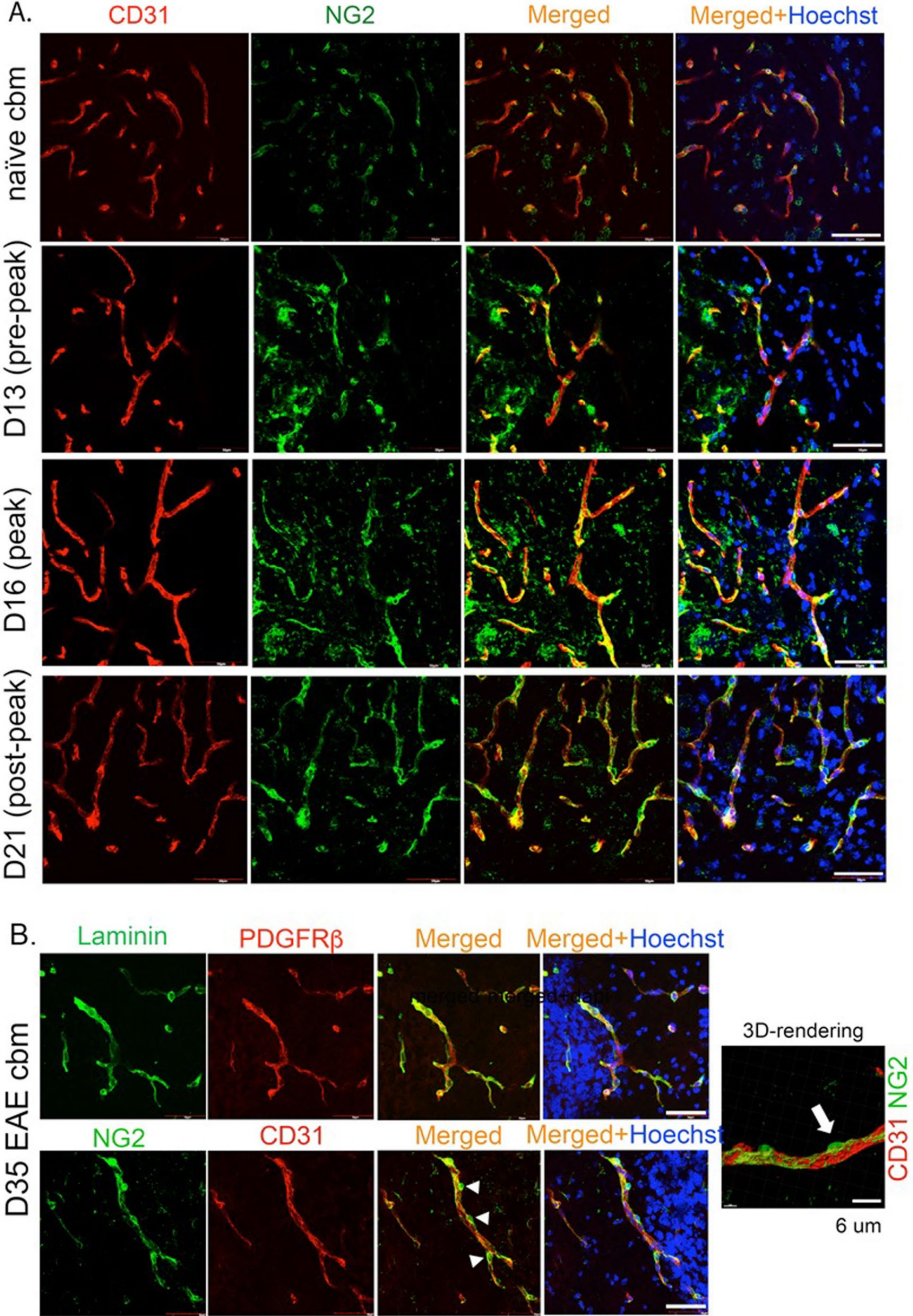
Confirmation of pericyte heterogeneity using NG2. **A** Pericytes share markers with other cell type, therefore, to rule out redundancy in the identification of pericytes, we also stained pericytes with NG2 and tracked them along the CD31+ endothelium. Similar to PDGFRβ+ cells, NG2+ pericytes look hypertrophied and elongated across all the time points in EAE cerebellar white matter. Scale 50 µm **B** Laminin/PDGFRβ and NG2/CD31 staining in chronic EAE (D35) reaffirming the attachment of pericytes to the NVU in later stages of EAE. Scale 50 µm. **A** 3D rendering using the Imaris image reconstruction software confirms the presence of NG2+ pericytes along the CD31+ vasculature. Scale 6 µm

EAE pathology affects white matter tracts in spinal cord and cerebellum tissues. Due to enlarged post-capillary venules and less diffused lesions in cerebellum [17], we investigated this CNS region for EAE-associated pathology. To study pericytes, we stained the sagittal cerebellar tissues from EAE (Fig. 1A) and naïve mice for PDGFRβ and NG2 expression (Figs. 1 and 2). The location of PDGFRβ+ and NG2+ cells within the vessels was evaluated by staining for pan-laminin, which delineates the basement membranes of vessels [17, 19] and by endothelial CD31 staining, respectively. Figure 1B shows laminin-delineated inflamed vasculature, used to examine the location of pericytes across pre-peak (days 10–13), peak (day 16), and post-peak (day 21) EAE tissue. At all the time points examined, PDGFRβ+ cells were found to be either encased or in close proximity to the pan-laminin+ basement membrane and infrequently noted in the parenchyma (Fig. 1A). Certain fields-of-view did appear to exhibit PDGFRβ+ cells away from the endothelial cells (post-peak; day 21), but a close examination revealed this observation to be due to weaker laminin staining in those images. Further, PDGFRβ+ cells were noted to be within the confinements of NVU as confirmed by GFAP+ reactive astrocytes in the peak EAE white matter (Additional file 1: Fig. S1).

These findings were corroborated by NG2+ pericytes, which were also found abutting the CD31+ endothelium (Fig. 2A). Even in chronic (day 35) EAE (Fig. 2B), pericytes detected by PDGFRβ or NG2 were vessel-associated and not encountered in the parenchyma. 3D-reconstruction of NG2+ cells in day 35 EAE white matter confirmed these cells to wrap around the CD31+ endothelium (Fig. 2B). It is noteworthy that NG2 is also a marker for oligodendrocyte precursor cells (OPCs), and the NG2+ cells away from CD31, as observed in some panels, could be representative of OPCs. Interestingly, NG2+ cerebellar pericytes that wrapped around CD31+ vasculature in the white matter of EAE appeared to be more elongated and interconnected compared to pericytes in naïve cerebellum (Fig. 2). These findings were consistent across all the analyzed time points within EAE cerebellar tissues. This morphological difference between EAE and naïve cerebellar tissues was quantified by calculating a pericyte coverage ratio, which is the ratio of pericyte-covered distance over total length of vasculature (described in methods; Fig. 1C). The pericyte coverage ratios of EAE capillaries were 0.635 ± 0.023, 0.646 ± 0.036, and 0.686 ± 0.017 during pre-peak, peak and post-peak EAE, respectively, which were significantly higher than the 0.334 ± 0.011 coverage ratio for naïve vasculature (Fig. 1C).

We then assessed pericyte density to determine if the difference in pericyte morphology was reflective of a change in the number of pericytes along the blood vasculature in EAE. Indeed, the pericyte density of 7.33 ± 0.58, 8.78 ± 0.49, 9.86 ± 1.16 during pre-peak, peak, and post-peak EAE, respectively, was significantly lower than the 19.70 ± 1.3, pericyte density observed in naïve condition (Fig. 1D). It is unclear whether pericytes underwent cell death that resulted in the loss of pericyte density as we did not observe any active caspase-3+ pericytes along the vasculature across all the time points (data not shown). Thus, despite a lower number of pericytes in EAE brain vasculature, these pericytes were elongated seemingly to extend coverage of the vessel to maintain its integrity.

### CSPGs as potential pericyte activators

**Fig. 3:**
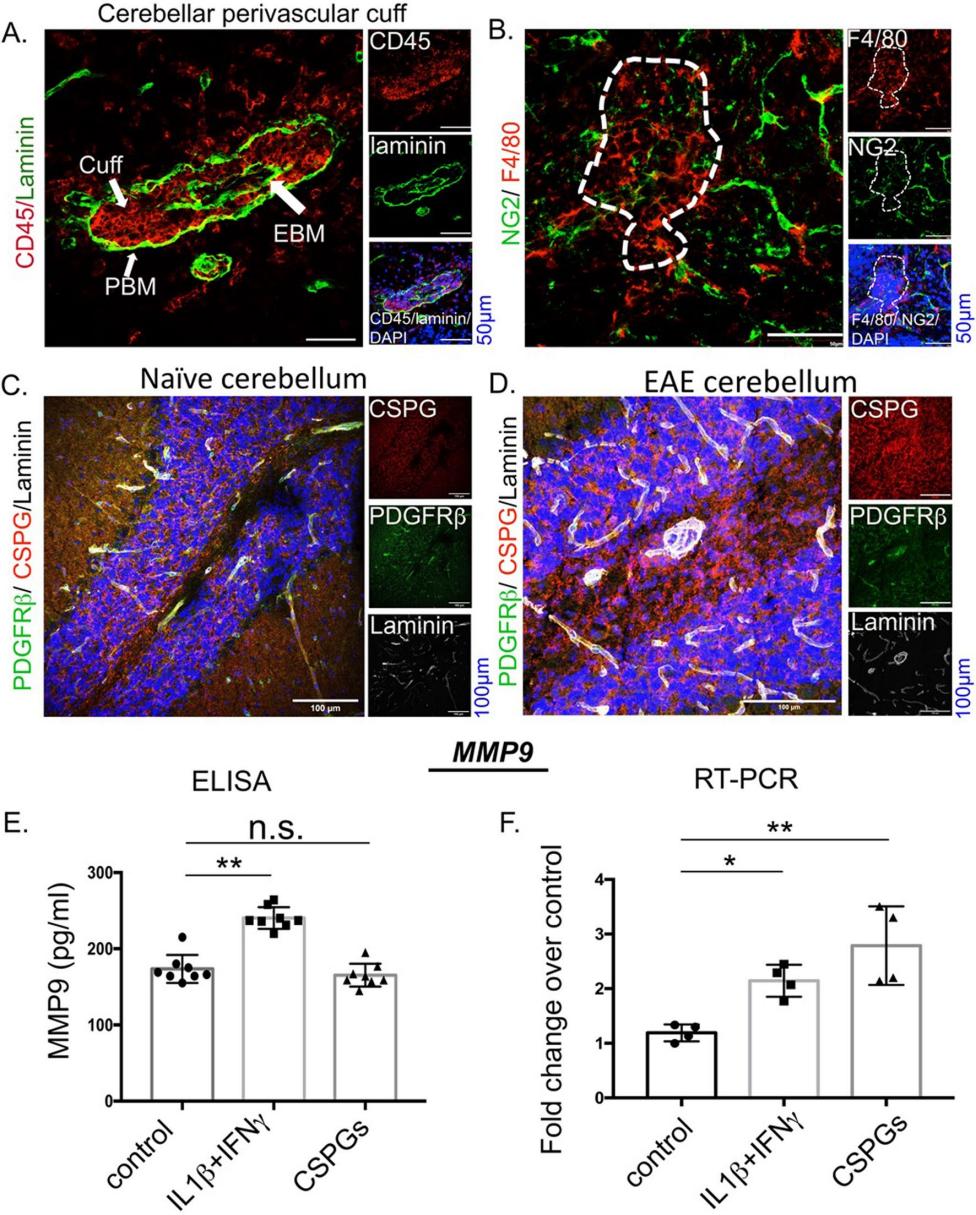
CSPGs as potential activators of pericytes. **A** A perivascular cuff from D16 EAE cerebellar white matter exhibiting laminin+ basement membranes (EBM-endothelial basement membrane; PBM-parenchymal basement membrane) laden with CD45+ leukocytes, majority of which are F4/80+ macrophages infiltrating the CNS (**B**). The selected region in B represents area corresponding to a perivascular cuff. Scale 50 µm. **C**, **D** Scale 50 µm. CSPG staining in naïve cerebellum (**C**) and around the cuffs in EAE cerebellum in close proximity to PDGFRβ+ pericytes (**D**). Scale 100 µm **E** MMP9 ELISA showing expression of MMP9 in culture medium from pericytes treated with 10 ng/mL each of IL-1β and IFN-γ, and 10 µg/mL of CSPGs. **F** RT-PCR of MMP9 transcripts in cultured pericytes in response to 6 h of stimulation with the inflammatory cocktail or CSPGs. Each datapoint in **E** and **F** represents technical replicates from different treatment conditions. Data are represented as mean ± SD. *p < 0.05, **p < 0.01

Due to their critical location along the BBB, pericytes are one of the first cellular components of the NVU to interact with leukocytes attempting to cross into the CNS from the circulation. To study this, we focused on perivascular cuffs, marked with laminin+ endothelial and parenchymal basement membranes separated by infiltrating CD45+ leukocytes (Fig. 3A). We noted that NG2+ pericytes in EAE were proximal to F4/80+ macrophage infiltrating brain regions (Fig. 3B). Moreover, in line with previous observations that CSPGs are elevated within and outside of EAE perivascular cuffs [16], we found widespread CSPG staining in EAE tissue including within the perivascular cuffs demarcated by laminin+ stain and PDGFRβ+ cells, when compared with the naïve cerebellum tissues (Fig. 3C, D). This suggests the potential of interactions between CSPGs and pericytes.

Pericytes produce matrix metalloproteinases-2 and -9 (MMP2 and 9) which cleave basement membrane constituents such as collagen IV, laminin, and fibronectin [20]. First, we addressed whether pericytes respond to different pro-inflammatory stimuli including CSPGs in culture. We treated murine pericytes in culture, which stained for both NG2 and PDGFRβ (Additional file 1: Fig. S3), with either CSPGs or a mixture of IL-1β and IFNγ (Fig. 3E, F). We observed that while the inflammatory cocktail of IL-1β and IFγ elevated MMP9 levels in culture supernatants, CSPGs failed to enhance the MMP9 production (Fig. 3E). However, CSPGs potentially upregulated the pro-MMP9 transcripts 6 h post-treatment (Fig. 3F), suggesting that CSPGs may influence pericyte activity. Similar to pericytes in EAE tissues, we did not observe any significant change in the number of Ki67+ PDGFRβ+ cells in culture upon CSPG or cytokine cocktail treatment (Additional file 1: Fig. S2). This suggests that murine pericytes in culture do not undergo a proliferative cell phase to support their inflammatory program.

### Pericytes as mediators of inflammation

**Fig. 4:**
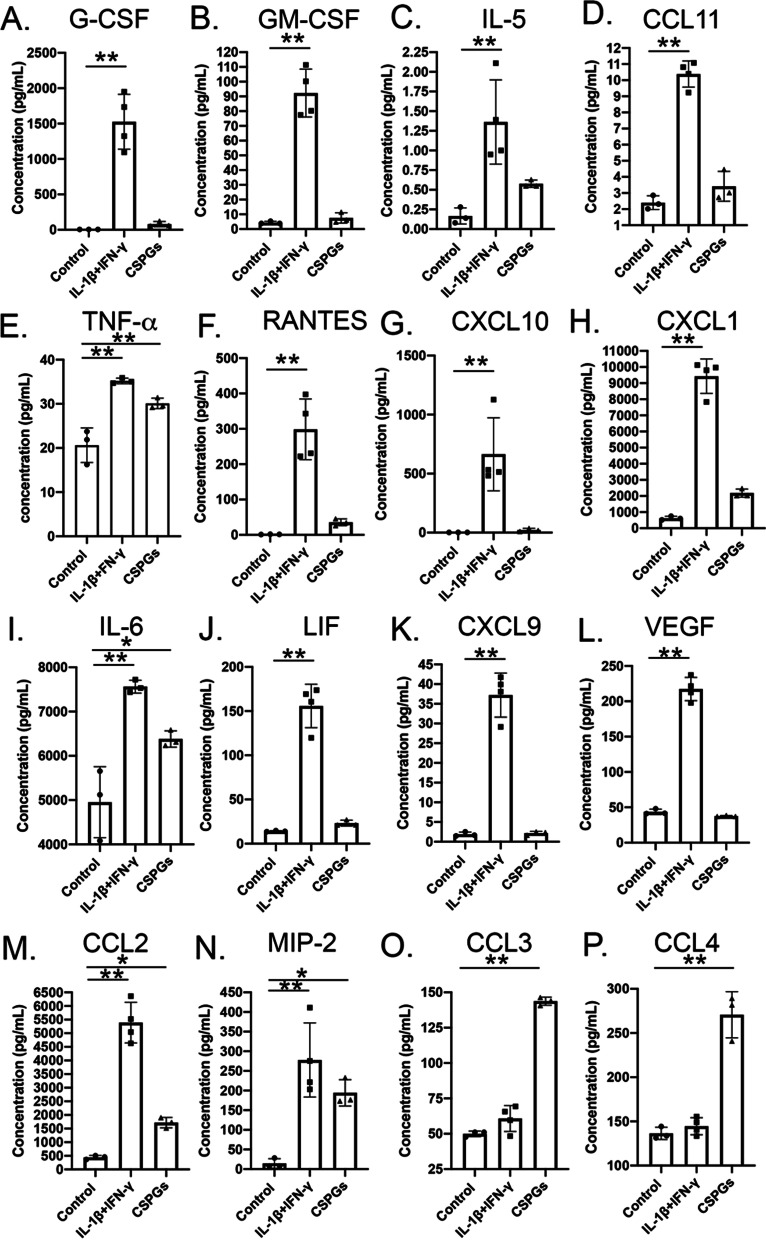
Inflammatory potential of pericytes. **A**–**P** Pericytes, when stimulated with the inflammatory cocktail of IL-1β and IFN-γ (10 ng/mL each), and CSPGs (10 µg/mL), secrete a plethora of cyto-chemokines, as exhibited by Multiplex ELISA (TNF-α was assayed separately using an ELISA kit). While G-CSF (**A**), GM-CSF (**B**), CCL11 (eotaxin) (**D**), RANTES (**F**), CXCL10 (IP-10) (**G**), CXCL1 (**H**), LIF (**J**), CXCL9 (**K**) and VEGF (**L**) are upregulated in response to IL-1β + IFN-γ, CSPGs specifically increased TNF-α (**E**), IL-6 (**I**), CCL2 (MCP-1) (**M**), and MIP-2, CCL3 and CCL4 proteins (**N**–**P**) in the cultured pericytes. Concentrations are expressed as pg/mL. Data are represented as mean ± SD. *p < 0.05, **p < 0.01

Pericytes secrete several adhesion molecules and chemokines/cytokines that assist in the recruitment and migration of monocytes, T cells, eosinophils, and neutrophils [21–23]. Pericytes also express pro-inflammatory factors such as IL-1β and TNFα, which can induce pro-inflammatory states in astrocytes, microglia, and endothelial cells, and help recruit leukocytes [20, 24]. Therefore, we studied the chemokine/cytokine profile of the pericyte secretome in response to inflammatory cytokines (IFN-γ + IL-1β) and CSPGs. Stimulation of pericytes with IFN-γ + IL-1β significantly upregulated the production of several inflammatory cytokines including granulocyte-colony stimulating factor (G-CSF; Fig. 4A), granulocyte–macrophage colony stimulating factor (GM-CSF; Fig. 4B), IL-5 (Fig. 4C), IL-6 (Fig. 4I), leukemia inhibitory factor (LIF; Fig. 4J), and vascular endothelial growth factor (VEGF; Fig. 4L). Chemokines including CCL11 (eotaxin; Fig. 4D), and chemokine (C-X-C) motif (CXCL1; Fig. 4H and CXCL9; Fig. 4K) were also upregulated in response to the treatment with IFN-γ + IL-1β (Fig. 4). Treatment with CSPGs upregulated pro-inflammatory cytokines, TNFα (Fig. 4E) and IL-6 (Fig. 4I) and CCL2 (Fig. 4M), a prominent chemotactic stimulus, for peripheral monocytes to transmigrate the inflamed CNS. Notably, the upregulation of CCL3 (macrophage inflammatory protein 1a; MIP-1a; Fig. 4O), and CCL4 (macrophage inflammatory protein 1b; MIP-1b; Fig. 4P) was significantly higher in response to treatment with CSPGs as compared to treatment with IFN-γ + IL-1β. This is critical since both these chemokines have been found to be elevated in the CSFs of relapse remitting MS, an acute inflammatory disease phase [25], suggesting a potential involvement of pericytes during inflammation in MS.

**Fig. 5:**
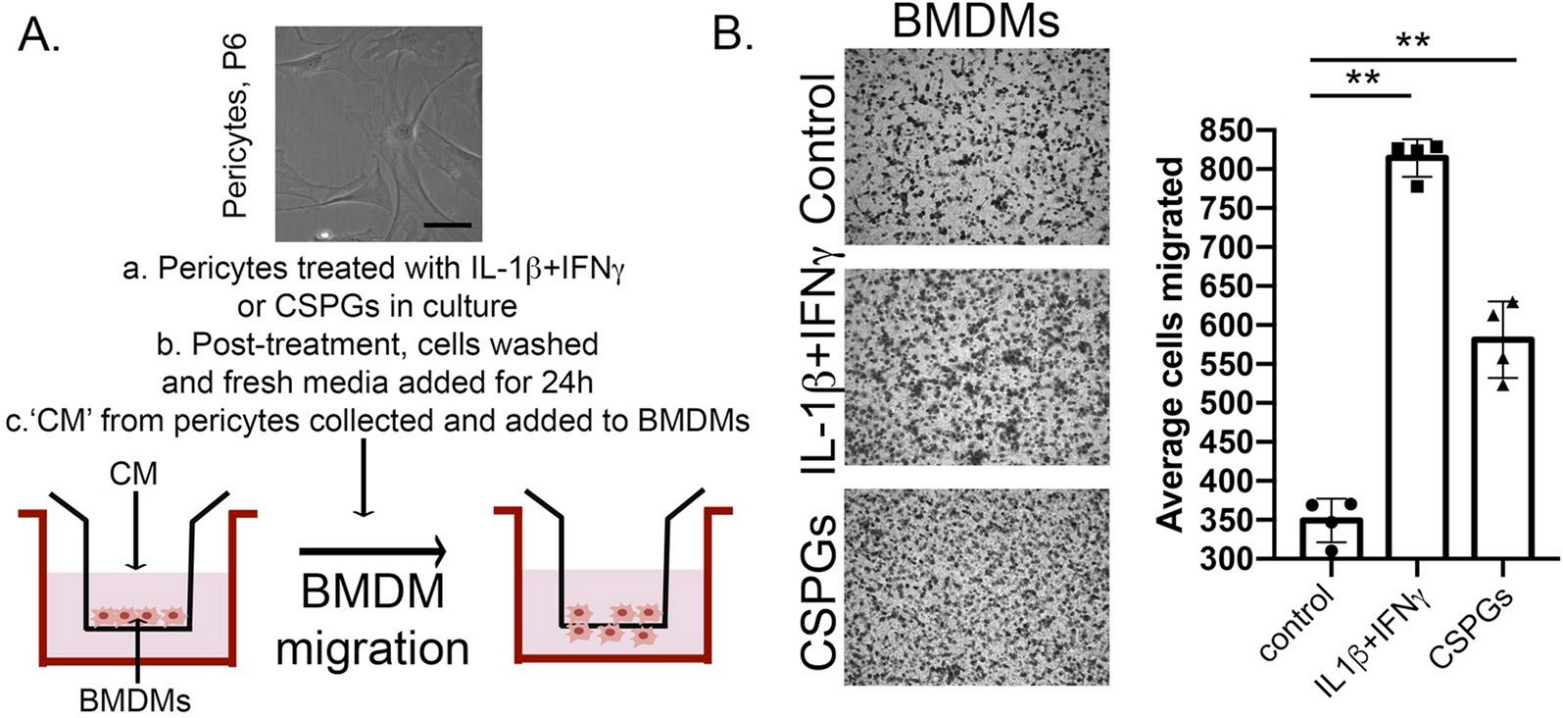
Inflamed pericytes facilitate transmigration of macrophages in vitro. **A** Using the Boyden chamber assay, we treated bone marrow derived macrophages (BMDMs) with culture media (CM) from either 10 ng/mL IL-1β + IFN-γ or 10 µg/mL CSPG-treated pericytes and compared with the potential of untreated pericyte media. **B** Brightfield images of fixed membranes showing BMDMs treated with the inflammatory cocktail as well as the CSPGs transmigrated significantly higher than the BMDMs from untreated pericytes, which is quantified in histograms to the right. Data are represented as mean ± SEM of quadriplicate analyses. **p < 0.01

Finally, to investigate whether the pericyte secretome in inflammatory conditions indeed has the potential to facilitate macrophage migration into the CNS parenchyma, the transmigration of BMDMs was assessed in vitro, using a Boyden chamber assay (Fig. 5A). When BMDMs were treated with conditioned medium from pericytes that were treated with inflammatory cocktail (IL1β + IFNγ), CSPGs (Fig. 5B) and LPS (Additional file 1: Fig. S4), there was significantly higher macrophage migration across the chamber as compared with controls stimulated with conditioned medium from untreated pericytes (Fig. 5B and Additional file 1: Fig. S4).

### Pericytes in MS lesions

**Fig. 6:**
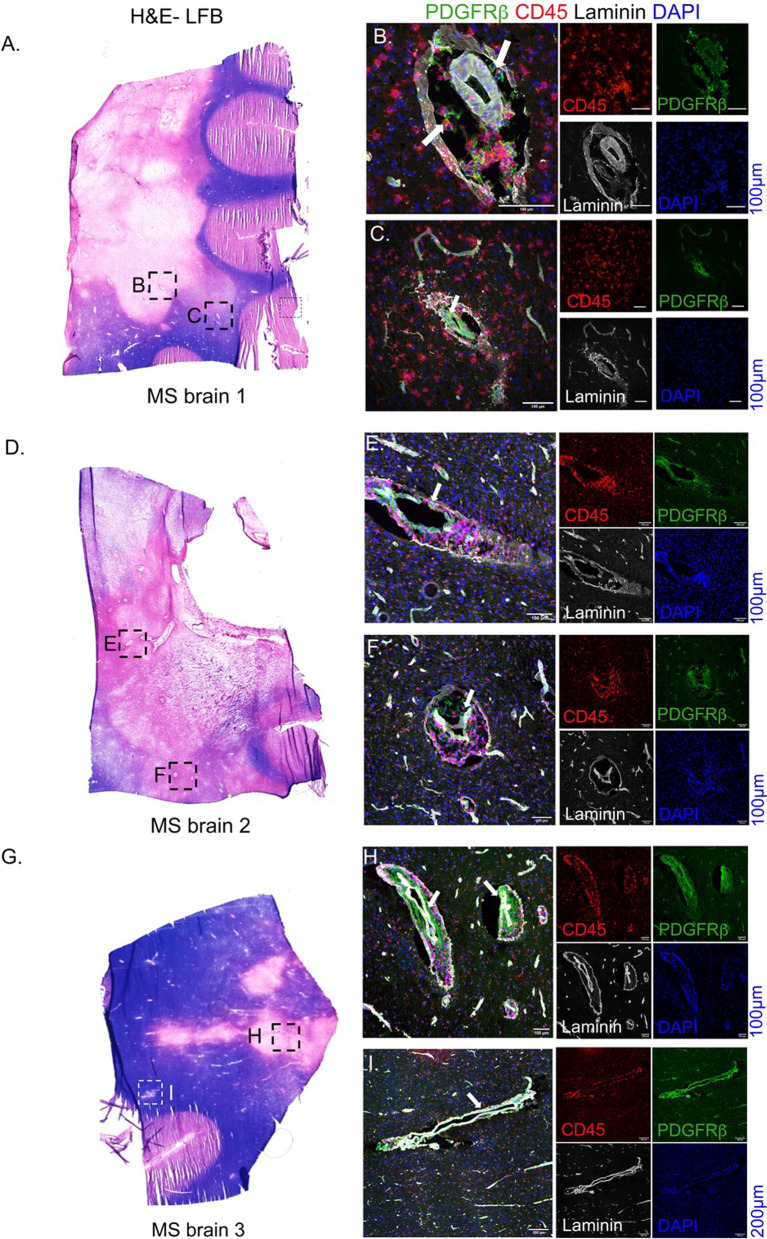
Pericytes in MS brain. Fresh-frozen MS brains (20 µm thick) harboring active lesions were investigated for pericyte morphologies. **A** H&E-luxol fast blue (H&E-LFB) staining of an MS brain section from a 60-year-old female showing demyelinating lesions. **B**, **C** MS brain section was stained for PDGFRβ and CD45 to locate the pericytes and infiltrating leukocytes in two different lesions demarcated as B and C in panel **A**. **D**–**F** MS tissue from a 61 year old male showing perivascular cuffs (CD45+ cells within laminin+ blood vessel boundaries) and intact PDGFRβ+ cells along the inflamed vasculature in two different brain regions, E and F. **G** Brain section from a 26 year old MS male also exhibits largely intact PDGFRβ+ staining encased within laminin+ basement membranes (**H** and **I**). Arrows show perivascular location of PDGFRβ+ pericytes in 2 regions with CD45+ leukocytes. Scale 100 µm

To identify if pericytes exhibit different morphologies and dynamics in MS brains, we investigated the location and morphology of PDGFRβ+ pericytes in lesions of 3 MS cases (Fig. 6A, D, G). We found PDGFRβ+ cells within the perivascular spaces as well as in close proximity to the endothelial barriers in all the MS lesions (Fig. 6B, C, E, F, H, I; shown with arrows). In all the fields, pericytes were found to be closely associated with laminin+ basement membranes. Notably, PDGFRβ+ cells were not detected within the parenchyma of MS brains even in proximity to highly inflamed vessels.

## Discussion

In normal physiological states, pericytes are known to play a role in BBB integrity by maintaining tight junctions and ensuring specific endothelial vesicular transport [8, 26]. During disease states, pericytes have been shown to migrate to the site of injury [27]. For instance, in traumatic brain and spinal cord injury, pericytes proliferate and ultimately outnumber astrocytes, and contribute to the scar formation by depositing ECM proteins [10]. In contrast to these findings, cerebellar pericytes in EAE appear to retain their microvascular location and encasement within the basement membrane. It is noteworthy that some PDGFRβ+ cells in the post-peak EAE appeared to be outside the basement membranes, but those regions have weaker laminin staining leading to such impressions. Also, some NG2+ cells appear outside CD31+ endothelial lining (Fig. 2) and it is highly likely that some of these cells represent OPCs or the staining potentially reflects non-specific staining. We corroborated our observations using MS brains. In our study, we noted PDGFRβ+ cells to be localized within the perivascular spaces in MS brain sections, where they appear not to be migrating to the parenchyma. If the pericytes had migrated, it is likely that we might not have captured those cells either due to differences in lesion chronicity or due to a limited field of view in MS brains. However, based on observations from multiple lesions from 3 different MS brains, we believe that our findings in MS are in contrast to the published reports suggesting migratory behavior of pericytes in different CNS conditions. In the light of these observations, immune-mediated component of EAE and MS needs to be taken into consideration to better understand the observation of non-migratory pericytes and the implication of this on immune cell extravasation into the CNS parenchyma. Further work is needed to investigate whether pericyte migration is elicited at a later time point in EAE or if there is pericyte migration into the parenchyma within the spinal cord or other areas of the brain besides the cerebellum in EAE, as pericyte might have different functions based on their anatomical location within the CNS [11].

This is one of the earlier studies to characterize morphological differences in pericytes within the EAE model as compared to naïve, with the changes starting as early as onset of EAE and persisting into the later phases of the EAE disease course. In line with our findings on pericyte density, Berthiaume et al. observed dynamic pericyte remodeling along the vasculature in response to the targeted ablation of neighboring pericytes [28]. Our findings of a decrease in pericyte density and an increase in coverage ratio in EAE suggests that the morphological change observed in EAE may be a compensatory mechanism in response to loss of pericyte density. Decreased pericyte density along the vasculature has been reported in other CNS disease states such as Alzheimer’s disease and stroke [29, 30]. Since pericytes that are lost along the vasculature do not appear to migrate into the parenchyma, the activation of death pathways in these pericytes need to be investigated to understand the mechanisms behind the loss in pericyte density in EAE cerebellum. In this regard, the activation of apoptotic pathways has been observed in a mouse model of stroke, where capillary pericyte loss was also observed in ischemic conditions [29]. However, our initial findings did not show caspase 3 activation in pericytes in vivo, however, other modes of death cannot be ruled out.

One of the key highlights of our studies is the upregulation of several cytokines and chemokines by pericytes in response to the inflammatory molecules, IFN-γ + IL-1β, indicating their ability to actively respond to and propagate inflammation. A previous study in our lab has demonstrated the ECM components, CSPGs, are novel mediators of leukocyte migration into the CNS by their capacity to upregulate motility and the secretion of a number of pro-inflammatory cytokines in macrophages [16]. CSPGs also induce the generation of pro-inflammatory chemokines in pericytes, which suggests a role for pericytes in facilitating the chemoattraction of monocyte/macrophages into the CNS. These findings are significant because they highlight the potential for dynamic interactions between ECM components, such as CSPGs and pericytes in mediating neuroinflammation. The secretions from pericytes stimulated with molecular mediators of inflammation such as IFN-γ + IL-1β and CSPGs upregulated the transmigration of BMDMs. It is also to be kept in mind that pericytes cultured in absence of endothelial cells may have behaviors that are not representative of their functions in vivo; however, we contend that our findings still shed light on the inflammatory potential of pericytes.

While it appears that pericytes have the capacity to indirectly induce leukocyte migration through their inflammatory secretome, it is yet to be determined if pericytes can directly facilitate leukocyte extravasation into the CNS. Pericytes have been observed to actively recruit and facilitate neutrophil migration in the study of inflammation in cremaster muscles [31, 32]. Live imaging shows the preferential migration of neutrophils through these gaps between pericytes, which was further facilitated by the expression of adhesion molecules by pericytes. The extent to which changes in pericyte morphology and secretome can directly facilitate immune cell recruitment and extravasation into the CNS parenchyma needs to be better understood.

## Conclusion

The ability of CSPGs to elicit pro-inflammatory responses in pericytes opens a new avenue for understanding the immunomodulatory functions of the ECM in MS. The findings of this study reinforce the potential role of two novel players in neuroinflammation, pericytes and CSPGs. Further investigation of the dynamic interactions between pericytes, CSPGs, and macrophages can help elucidate the mechanisms of immune cell extravasation into the CNS in the context of inflammatory conditions such as MS.

## Supplementary Information

**Additional file 1: Figure S1.** Pericytes localized within GFAP+ reactive astrocytes in EAE. D16 EAE cerebellum (cbm) tissues (lower panel) were analyzed for GFAP+ astrocyte (green) and PDGFRβ+ pericyte (red) staining to investigate differences in these cells as a part of the neurovascular unit (NVU). When compared with naïve cerebellum (upper panel), reactive astrocytes in EAE cerebellum appear to wrap around the PDGFRβ+ cells in an inflamed capillary venule. Scale 50 µm. **Figure S2.** Pericyte proliferation is not altered upon IL-1β and IFNγ or CSPG treatments. A. Primary mouse pericytes were seeded at 7,500 cells in a 96-well plate and treated with either 10 ng/mL IL-1β + IFNγ or 10 µg/mL CSPG and stained for DAPI and Ki67 to identify proliferating cells. **B.** Graphs denote %Ki67+ DAPI+ cells in response to treatment. Data are represented as mean ± SD. **Figure S3.** Characterization of mouse pericytes in vitro. Primary murine pericytes (passage 6) were seeded at 7500 cells in 96-well plates and stained for PDGFRβ (red) and NG2 (green) after 24 h in cultures. These cells were found to express both these markers. **Figure S4.** Pericyte induced macrophage migration in vitro. Using the Boyden chamber assay, we investigated migration of bone marrow derived macrophages (BMDMs) in response to supernatants from LPS-treated pericytes. Data points represent technical replicates in untreated and LPS-treated conditions. Data are represented as mean ± SD. **p < 0.01.

## Data Availability

All data generated or analyzed during this study are included in this published article and its Additional files.

## Abbreviations

MS: Multiple sclerosis
CNS: Central nervous system
BBB: Blood–brain barrier
EAE: Experimental autoimmune encephalomyelitis
NVU: Neurovascular unit
ECM: Extracellular matrix
CSPGs: Chondroitin sulfate proteoglycans
OPC: Oligodendrocyte precursor cells
MOG: Myelin oligodendrocyte glycoprotein
PDGFRβ: Platelet-derived growth factor-β
NG2: Neural/glial antigen 2
MMP9: Matrix metalloproteinase inducer-9
IFNγ: Interferon-γ
IL-1β: Interleukin-1β
BMDM: Bone marrow-derived macrophages
CD45: Cluster of differentiation CD45
CD31: Cluster of differentiation CD31
LPS: Lipopolysaccharide
G-CSF: Granulocyte-colony stimulating factor
GM-CSF: Granulocyte-macrophage colony stimulating factor
LIF: Leukemia inhibitory factor
VEGF: Vascular endothelial growth factor
