# Supplementary figures and images for: Pericytes as mediators of infiltration of macrophages in multiple sclerosis
Source: J Neuroinflammation. 2021 Dec 24;18:301. doi: 10.1186/s12974-021-02358-x (PMC8705458; doi:10.1186/s12974-021-02358-x)

Fig S1

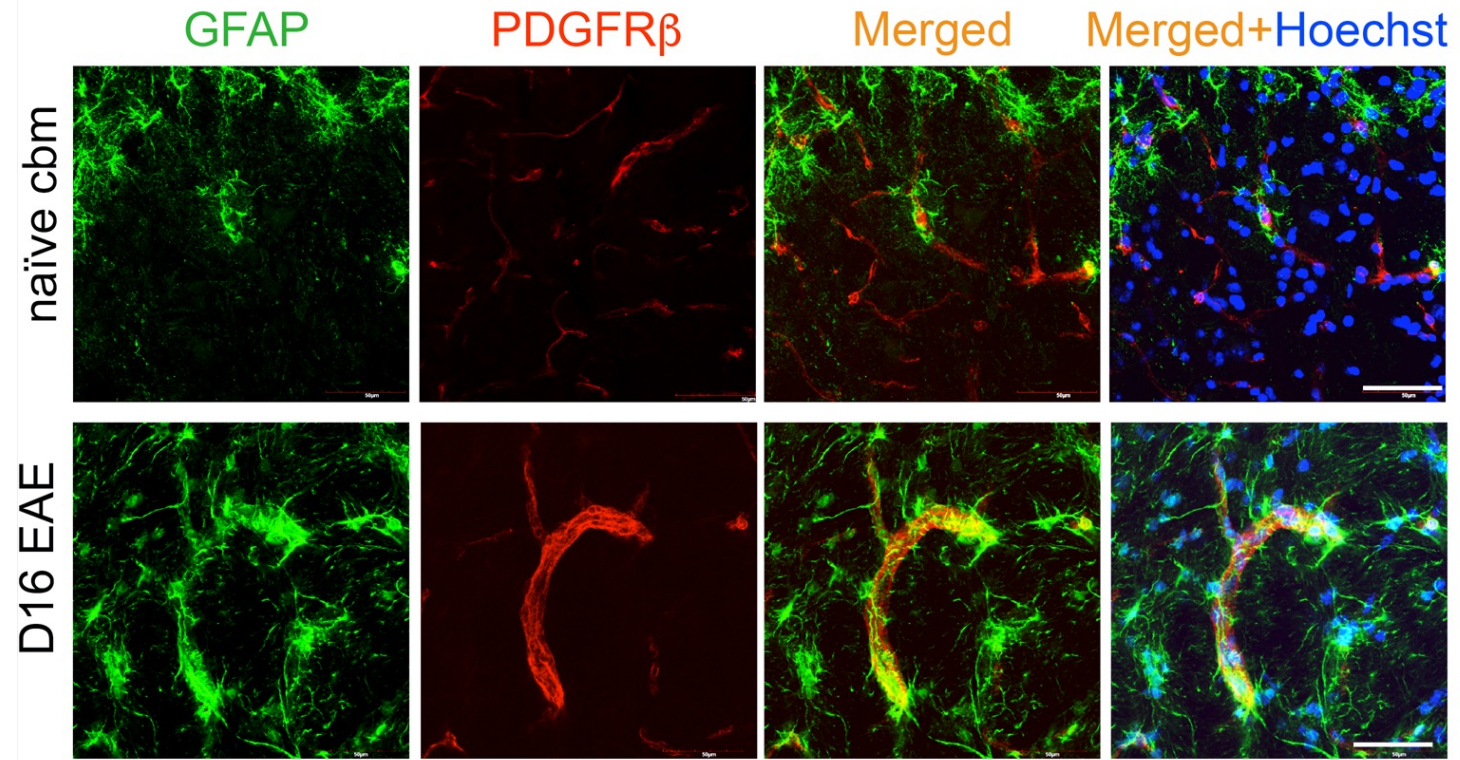

Fig S2

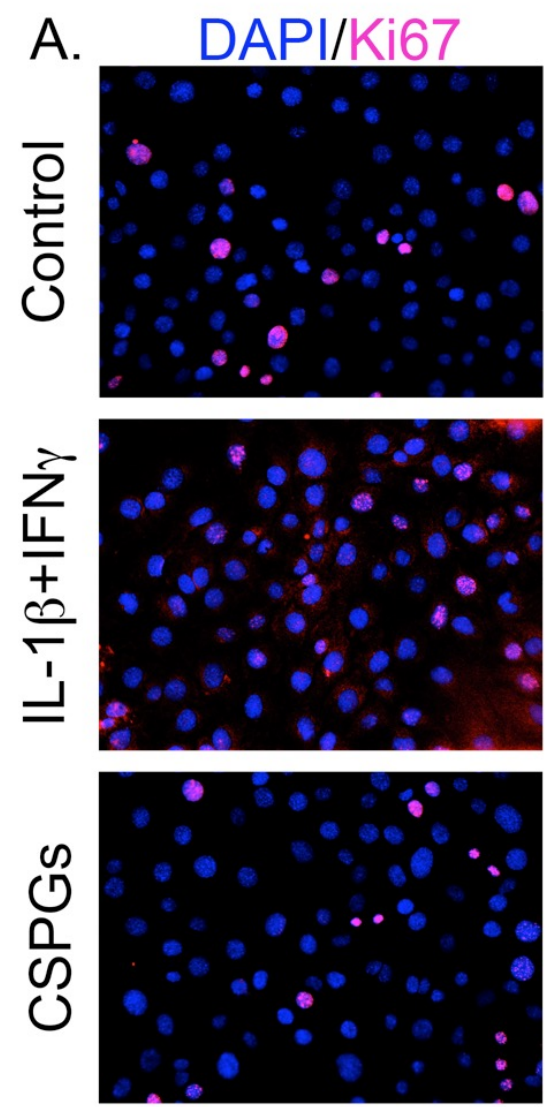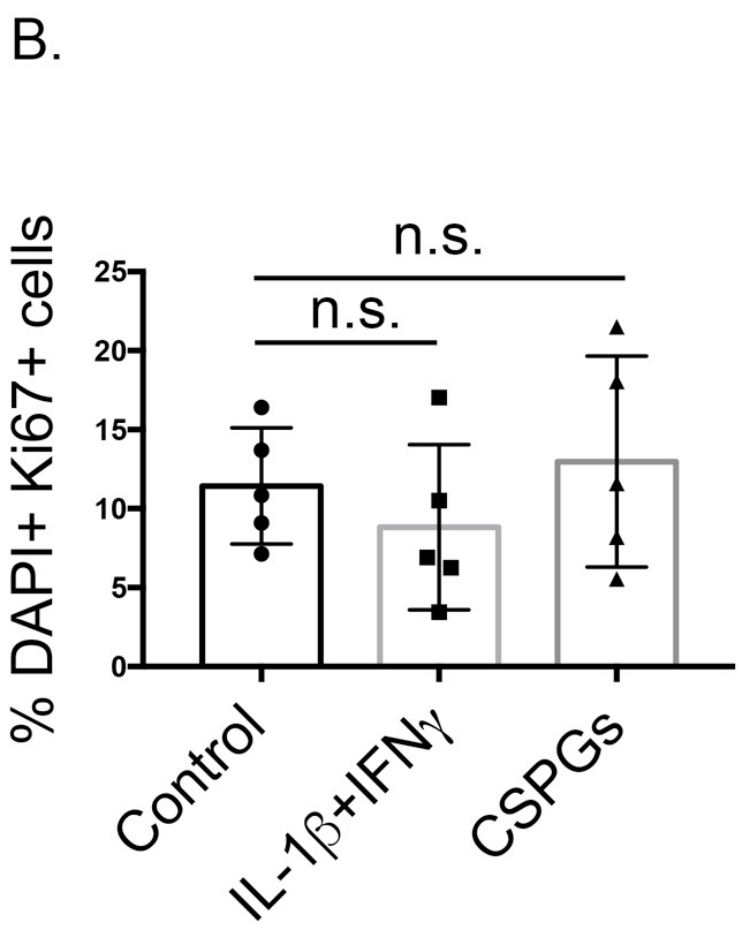

Fig S3

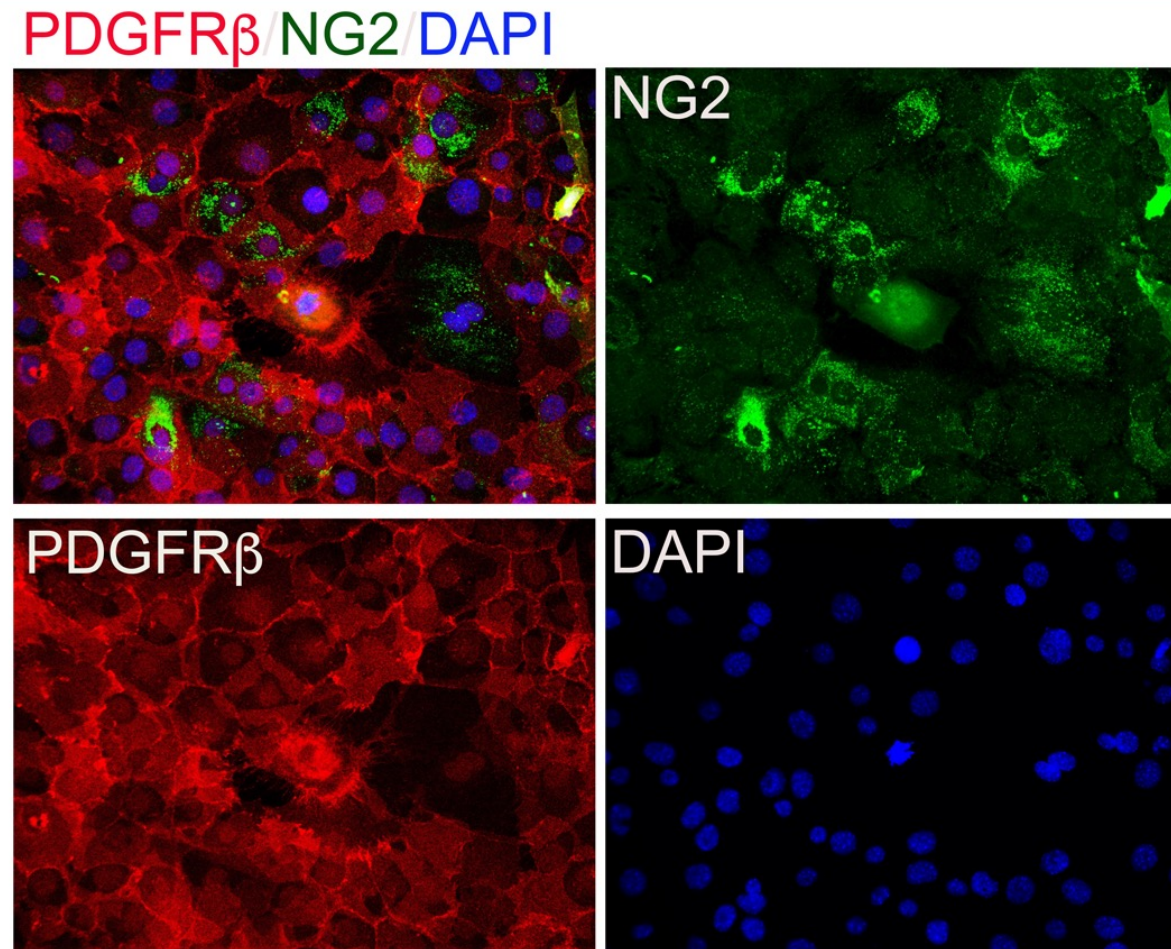

Mouse capillary pericytes in culture, P6

Fig S4

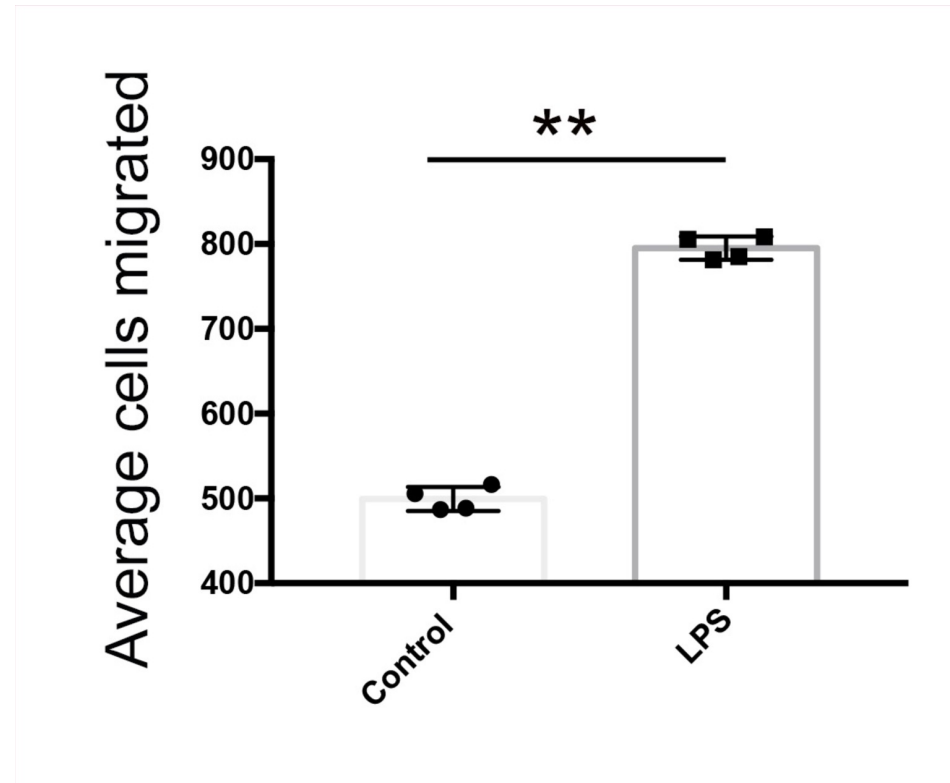

Supplement: Supplementary file 1 — Additional file 1: Figure S1. Pericytes localized within GFAP+ reactive astrocytes in EAE. D16 EAE cerebellum (cbm) tissues (lower panel) were analyzed for GFAP+ astrocyte (green) and PDGFRβ+ pericyte (red) staining to investigate differences in these cells as a part of the neurovascular unit (NVU). When compared with naïve cerebellum (upper panel), reactive astrocytes in EAE cerebellum appear to wrap around the PDGFRβ+ cells in an inflamed capillary venule. Scale 50 µm. Figure S2. Pericyte proliferation is not altered upon IL-1β and IFNγ or CSPG treatments. A. Primary mouse pericytes were seeded at 7,500 cells in a 96-well plate and treated with either 10 ng/mL IL-1β + IFNγ or 10 µg/mL CSPG and stained for DAPI and Ki67 to identify proliferating cells. B. Graphs denote %Ki67+ DAPI+ cells in response to treatment. Data are represented as mean ± SD. Figure S3. Characterization of mouse pericytes in vitro. Primary murine pericytes (passage 6) were seeded at 7500 cells in 96-well plates and stained for PDGFRβ (red) and NG2 (green) after 24 h in cultures. These cells were found to express both these markers. Figure S4. Pericyte induced macrophage migration in vitro. Using the Boyden chamber assay, we investigated migration of bone marrow derived macrophages (BMDMs) in response to supernatants from LPS-treated pericytes. Data points represent technical replicates in untreated and LPS-treated conditions. Data are represented as mean ± SD. **p < 0.01. [file 12974_2021_2358_MOESM1_ESM.pdf]
